# Better Management for Neuroendocrine Neoplasms: A Complex Task Ahead

**DOI:** 10.3390/jcm10091859

**Published:** 2021-04-25

**Authors:** Romain Coriat

**Affiliations:** 1 Gastroenterology and Digestive Oncology Unit, Cochin Hospital, AP-HP Centre, 27 rue du Faubourg Saint-Jacques, 75014 Paris, France; romain.coriat@aphp.fr; Tel.: +33-1-58-41-19-52; 2 UFR de Médecine, Faculté de Santé, Université de Paris, 75006 Paris, France

Neuroendocrine neoplasms (NENs) are rare and indolent tumors characterized by the ability to synthesize, store, and secrete a variety of neuro-amines and peptides which can result in a secretory syndrome. Their incidence has increased in the past three decades in the context of improvement and development of both radiological and endoscopy explorations [1]. 

NENs are clinically heterogeneous tumors with an ability to develop distant metastasis. It has recently been highlighted that, over the past three decades, the overall survival of NENs improved, especially for distant-stage gastrointestinal and pancreatic neuroendocrine lesions.

A tipping point in NENs management was reached in 2010 with the publication of the World Health Organization (WHO) classification which identified different categories of NENs according to their potential aggressiveness [2]. NEN grouped Neuroendocrine tumors (NET) and Neuroendocrine Carcinoma (NEC). The 2010 WHO classification was based on cell differentiation, number of mitosis and Ki-67 proliferation index. The distinction between Grade 1, 2 or 3 tumors relied on both mitotic index and Ki-67 proliferation indexes. An update of the 2010 WHO classification was first published in 2017 for pancreatic lesions and later validated for all digestive sites in 2019. We now consider two types of digestive NENs: well-differentiated NET (Grade 1, 2 or 3) and poorly differentiated tumors (Grade 3 NEC). Despite this recent update, the management of NENs remains complex and multimodal. 

As mentioned above, the 2017 WHO classification added a new category, the well-differentiated grade 3 NETs. Management of these grade 3 NETs remains a challenge considering the aggressiveness of lesions with a Ki-67 index above 20% [3]. The main localization of grade 3 NETs is the pancreas. Treatment for grade 3 NETs is not fully standardized and differs from lower-grade NETs or NEC treatment. Herein, Pellat et al. give overall guidelines for the management of grade 3 NETs and discussed the new therapeutic options for this rare entity [4,5].

One of the cornerstones of NENs management is surgery, irrespective of tumor site (for both pancreatic and midgut tumors). In the present Special Issue, Souche et al. stress the surgical management of pancreatic NETs according to staging. They describe all the required steps, starting with a comprehensive preoperative evaluation, to allow for the best surgical approach (open versus laparoscopic, standard versus sparing parenchymal pancreatectomy, lymphadenectomy). Among other things, they discuss strategies to enhance the short- and long-term benefit/risk ratio in pancreatic NET patients [6]. Deguelte et al. focus on the surgical approach of small-intestinal NETs, which plays a key role in the multimodal management of these lesions [7]. Small-intestine NETs are mainly diagnosed at a metastatic stage and curative intent with an R0 resection is feasible in less than 20% of cases. Nevertheless, even in the metastatic setting, resection of the primary tumor +/− metastasis should be discussed to avoid abdominal symptoms (such as occlusion), reduce hormonal secretion and/or decrease tumor volume. As stressed by Deguelte et al., surgeons must be aware of the specific surgical management of small intestinal NETs including the need for bowel-preserving surgery to avoid short bowel syndrome. 

The medical management of advanced NETs has reached a certain level of efficacy. In the past decade, several phase III studies have validated new therapeutics options for NETs such as targeted therapies (Tyrosine kinase inhibitors, mTOR inhibitor, somatostatin analogs) and peptide receptor radionuclide therapy [8,9,10,11]. These new therapeutic approaches are also discussed and positioned in two dedicated articles of this Issue [12,13]. 

Finally, the recent development of interventional radiology has offered a new therapeutic approach for patients with metastatic NETs. Radiological liver-directed therapies such as transarterial embolization, transarterial chemoembolization, and selective internal radiation therapy, play a crucial role in the management of NET patients with liver metastasis in order to improve symptoms and overall survival. In the present issue, Barat et al. present all new radiological options and discuss their position in the overall management of NETs [14]. 

The overall survival of patients with NETs is increasing with the development of new surgical, chemical, oncological, and radiological approaches. NETs require a multimodal approach, which should be discussed in dedicated multidisciplinary meetings, in order to propose the optimal therapy for each patient. 
